# Exogenous Melatonin Treatment Induces Disease Resistance against *Botrytis cinerea* on Post-Harvest Grapes by Activating Defence Responses

**DOI:** 10.3390/foods11152231

**Published:** 2022-07-27

**Authors:** Zezhen Li, Shujuan Zhang, Jianxin Xue, Bingyu Mu, Hong Song, Yanping Liu

**Affiliations:** 1 College of Agricultural Engineering, Shanxi Agricultural University, Jinzhong 030801, China; z15035658426@163.com (Z.L.); jx18235400069@163.com (J.X.); 13633544838@163.com (B.M.); 2 College of Food Science and Engineering, Shanxi Agricultural University, Jinzhong 030801, China; song150506721@163.com (H.S.); L15222112409@163.com (Y.L.)

**Keywords:** post-harvest grapes, grey mould, exogenous melatonin, *Botrytis cinerea*, induced resistance

## Abstract

*Botrytis cinerea* seriously affects the value of post-harvest grapes. Melatonin can act as an exogenous regulator in the resistance of exogenous pathogens due to its antioxidant activity. An artificial inoculation trial was conducted to research the induced resistance mechanism of melatonin treatment using the table grape “Muscat Hamburg” (*Vitis vinifera* L. cv). Grapes were immersed with 0.02, 0.2, and 2 mmol/L melatonin, followed by *B. cinerea* suspension injections after 48 h. The results showed that the mycelial growth and spore germination of *B. cinerea* was not significantly inhibited by melatonin at different concentrations (0.02–2 mmol/L). However, post-harvest melatonin treatment inhibited the increase of disease incidence and severity of grey mould, induced the synthesis and accumulation of total phenols and flavonoids, reduced malondialdehyde generation, and inhibited an increase in cell membrane permeability. Meanwhile, defensive enzyme activities, including superoxide dismutase (SOD), peroxidize (POD), catalase (CAT), phenylalanine ammonia-lyase (PAL), polyphenol oxidase (PPO), chitinase (CHI), and β-1,3-glucanase, were significantly increased in fruits treated with exogenous melatonin. These results suggested that exogenous melatonin treatment could activate defence responses to combat the infection of *B. cinerea* in post-harvest grapes.

## 1. Introduction

Grapes (*Vitis vinifera* L.) are one of the essential fruit crops globally. This is due to their rich nutritional value, such as high contents of vitamins, flavonoids, anthocyanins, and tannins [1,2]. However, grey mould is a major fungal disease in grapes, which can lead to heavy loss during pre- and post-harvest stages [3]. The pathogenic fungal of grey mould is *Botrytis cinerea*, which can be latent from the flowering stage to the post-harvest onset or can directly occur through a wound on the surface of the fruit. This pathogen spreads easily among grapes and can tolerate temperatures down to −0.5 °C [4]. According to reports, economic losses caused by grey mould on grapes are as high as 20–40% and even as high as 60% [5,6]. Therefore, studying the prevention and post-harvest control of grey mould is crucial.

At present, chemical fungicides are the primary control strategy for grey mould [7,8]. However, using chemical fungicides may result in high toxicity, biodiversity loss, and increased drug resistance in fungi [9,10,11,12]. Their growing adverse effects have led to the search for alternative disease-control strategies. The increasing research has shown that the defence responses of plants can be activated to combat microbial attacks. Plant diseases can be controlled by inducing resistance through chemical, physical, and biological methods. This may serve as a sustainable strategy to prevent post-harvest deterioration of plants [13] and is the most promising new disease-control technology to replace chemical fungicides, receiving increasing attention in recent years [7,14,15,16,17].

Melatonin (MT, chemical name: N-acetyl-5-methoxy-tryptamine) is released from the pineal gland as an endocrine hormone found in various organisms [18]. When MT was initially detected in plant tissues in 1995 [19], subsequent studies found that it is widely present in the various tissues of higher plants, in very low amounts. Usually, the order of magnitude of MT is pg/g–ng/g [20,21,22] in tissue samples. MT is considered to have important functions in biological and abiotic stress [23,24,25,26]. The increased content of endogenous MT could alleviate the damage caused by various environmental stresses, including biological factors such as pathogens and pests, as well as abiological factors such as high salinity, heavy metals, drought, and low temperature [22,27]. There have been studies showing that exogenous MT treatment significantly induced resistance to *Diplocarpon mali* in apple leaves [28]. Studies also stated that exogenous MT treatment could reduce post-harvest decay and maintain the nutritional quality of strawberries [29,30]. Cao et al. [31] indicated that exogenous MT induced the resistance to grey mould in apples by continuously increasing the defence enzyme activities in fruits. Li et al. [32] revealed that exogenous MT application could improve fruits’ disease resistance, particularly by inhibiting grey mould in cherry tomato fruits, increasing the level of defence-related enzymes and metabolites, and inducing the accumulation of endogenous MT and salicylic acid (SA). Sheng et al. [33] reported that pretreatment with MT could enhance the disease resistance of post-harvest tomato fruit, increase the related enzyme activities, and thus improve the storage quality. In addition, exogenous MT treatment in strawberries could inhibit the development of black spot diseases and enhance disease resistance [34].

At present, there has been increasing attention to exogenous MT treatment and elucidating the biocontrol mechanisms against post-harvest fruit grey mould caused by *B. cinerea*. However, there are no reports focused on grape disease resistance induced by MT. Accordingly, our study tried to explore the efficacy of exogenous MT treatment in inducing disease resistance of grapes against *B. cinerea*, and further investigated the possible mechanisms involved.

## 2. Materials and Methods

### 2.1. Materials and Treatment Methods

The table grapes “Muscat Hamburg” were harvested from an orchard in Taigu City, Shanxi, China (37°34′ N, 112°50′ E). Grapes with uniform maturity and shape, without injuries, were selected for experiments and transported to the laboratory within 2 h. Melatonin, L-phenylalanine, snailase, laminarin, colloidal chitin, and rutin were purchased from Sigma-Aldrich (Shanghai) Trading Co., Ltd. (Shanghai, China). Guaiacol, catechol, thiobarbituric acid, 3, 5-dinitrosalicylic acid, N-acetylglucosamine, gallic acid, and Folin-Ciocalteu were obtained from Beijing Solarbio Science and Technology Co., Ltd. (Beijing, China). In this study, all other chemicals were analytical grade and purchased from Sinopharm Chemical Reagent Co., Ltd. (Tianjin, China).

Grape berries with 0.2 cm pedicel were prepared and submerged in 1% (*v*/*v*) sodium hypochlorite for 2 min to sterilize them. Next, grape berries were washed in distilled water three times and divided into 5 groups of 240 berries each. Grapes in treated groups were soaked in a solution of 0.02, 0.2, or 2 mmol/L MT with 0.05% Tween-20 for 20 min and dried in air. The positive and negative control groups consisted of grapes immersed in distilled water. Then, grapes were sat in trays lined with sterile gauze immersed in water and covered with plastic wrap. After 48 h, a suspension of *B. cinerea* (10 μL, 10^6^ CFU/mL) was injected into the wound (3 mm deep) of grape berries, except for the negative control grapes. Then, the grapes were arranged in trays lined with sterile gauze immersed in water, covered with plastic wrap, and stored in the incubator (Beijing Kewei Yongxing, HWS-150, Beijing, China) at 21 °C and 95% relative humidity. All experimental treatments were replicated thrice. Grape berries (30 fruits) were taken on 0, 1, 2, 3, 4, 6, and 8 days after inoculation for determination of all indicators with three biological repetitions.

### 2.2. Pathogen

The pathogen *B. cinerea*, provided by the College of Plant Protection, Shanxi Agricultural University, was subcultured on potato dextrose agar (PDA) medium away from light (21 °C) as described by Lachhab et al. [35]. The spore suspension was prepared with *B. cinerea* spores removed from 10-day-old colonies, and the final concentration used for inoculation was 1 × 10^6^ CFU/mL and adjusted with sterile distilled water including 0.05% Tween-80 as measured by a hemocytometer.

### 2.3. Effect of MT on Pathogenic Fungi

#### 2.3.1. Effect of MT on Spore Germination of *B. cinerea* in Potato Dextrose Broth (PDB) Medium

In the 50 mL triangular flask, the final concentration of MT mixed with PDB medium was 0.02, 0.2, or 2 mmol/L. They were then inoculated with the pathogen spore suspension (1 × 10^6^ CFU/mL), and the triangular flasks were placed on a shaker (300 rpm) for 8 h. The spores were sampled and observed under a microscope with a ×40 objective (Olympus, CX23LEDRFS1C, Beijing, China). The germination tube length should equal at least half the spore length for the spore to be considered germinated. The germination of 200 spores was observed in each treatment and replicate, and the results were expressed as the average spore germination rate (%). There were three independent replicates per treatment.

#### 2.3.2. Effect of MT on Mycelial Growth of *B. cinerea* in PDA Medium

PDA medium containing 0.02, 0.2, or 2 mmol/L MT was prepared. The control medium was added with the same volume of sterile water. Then, pathogen spore suspension (50 μL, 1 × 10^6^ CFU/mL) was infused into a hole drilled with an Oxford cup in the middle of the plate and incubated. Four days later, the mean diameter of the fungal colony was measured using the crisscross method with callipers incubated at 21 °C. Each group had three plates, and experiments were repeated thrice.

### 2.4. Assays of Disease Incidence (DI) and Disease Severity (DS)

DI (%) was evaluated by the percentage of infected berries number compared to total berries inoculated with *B. cinerea* [36].

DS was evaluated by the extent of decay on the grapes: 0, healthy berry; 1, lesion area < 5%; 2, 5% ≤ lesion area < 25%; 3, 25% ≤ lesion area < 50%; 4, 50% ≤ lesion area < 85%; 5, lesion area ≥ 85%.

The following formula was used to calculate the DS:

DS = ∑ (level × number of grapes at each level)/5 × total number of berries(1)

### 2.5. Assay of Malondialdehyde (MDA) Content and Cell Membrane Permeability

Lipid peroxidation extent was estimated by MDA content measured by the thiobarbituric acid (TBA) method [37]. Grape tissue (1 g) was weighed, transferred to a mortar with trichloroacetic acid (TCA, 5 mL, 5%), and ground into a homogenate. After 10 min of low-temperature centrifugation using an Eppendorf 5805 centrifuge (Eppendorf AG, Hamburg, Germany) at 10,000× *g*, 2 mL supernatant was removed, added to 0.67% TBA (2 mL), and then placed in boiling water for 20 min. The solution was cooled prior to centrifugation (4 °C, 10,000× *g* for 10 min), and the absorbance (OD) values at 450, 532, and 600 nm were recorded using a UV-VIS spectrophotometer (Shanghai Youke, T2600, Shanghai, China). The result was averaged and expressed as nmol g^−1^ (fresh-weight basis).

The permeability of the cell membranes was evaluated by determining the relative electrical conductivity (REC). It was performed as Liu et al. described [38], with minor alterations. In a 100 mL beaker, 30 disc pieces (1 mm thick) cut from the 20 fruits were washed three times with deionized water for 30 s. Deionized water (50 mL) was added to the beakers with disc pieces and shaken for 30 min. C1 was determined for initial electrolyte leakage using a DDS-IIA conductivity meter (Leici, Shanghai, China). When the beakers were kept in water at 100 °C for 20 min and cooled down, the final electrical conductivity (C2) was recorded. The REC of grape berries was calculated using the following formula: REC (%) = C1/C2 × 100.

### 2.6. Assays of Total Phenolics and Flavonoids Content

Total phenolics and flavonoid compounds were extracted using a methanol–HCl solution (60% methanol, 0.1% hydrochloric acid) as described by Liu et al. [39]. Grape peels were separated from pulps, and a 2 g sample was crushed in a mortar and pestle cooled by liquid nitrogen. The powder was extracted with a 30 mL methanol-HCl solution (60% methanol, 0.1% hydrochloric acid) away from light in an ultrasonic water bath for 0.5 h (40 °C), filtered, and then centrifuged at 10,000× *g* for 10 min at 4 °C. Extraction was repeated thrice, and the combined supernatant was prepared to analyse total phenolics and flavonoid contents.

The Folin–Ciocalteu method was performed to determine total phenolic content as described by Sarneckis et al. [40]. A total of 0.4 mL of the extract was removed, and then Folin-Ciocalteu (5 mL, 10-fold dilution with distilled water), 1.5 mL of 20% sodium carbonate, and 3.1 mL of distilled water were added successively. After mixing well, the solution was placed at 20 °C, away from light for 2 h. The OD725 value was determined. Gallic acid served as the reference standard used to plot standard curves, and the results were calculated and presented as mg gallic acid equivalent per g of fresh sample (mg GAE/g).

The aluminium colourimetric method was used to measure total flavonoid content [41]. A total of 0.4 mL of the sample supernatant was added to the beaker and mixed with 0.6 mL of methanol (100%) as well as 2.7 mL of 30% methanol. Then 0.2 mL of nitrite (0.5 mol/mL) and 0.2 mL of chloride hexahydrate (0.3 mol/L) were added to the mixture successively and mixed well. Finally, sodium hydroxide (1.0 mL, 1 mol/L) was vortex-mixed with the solution, which was placed for 5 min, and measured at OD510 nm. The results were calculated from standard curves plotted using rutin (0–100 µg/mL), and presented as mg rutin equivalent per g of fresh samples (mg CE/g).

### 2.7. Assays of Antioxidant Enzyme Activity

Evaluation of superoxide dismutase (SOD) activity was conducted as protocols of the SOD assay kit (A001-1; Jiancheng Corp., Nanjing, China). A total of 10% tissue homogenate consisting of a 1 g sample and 9 mL phosphate buffer (pH 7) was prepared and centrifuged (4 °C, 10,000× *g* for 10 min) to collect the supernatant for SOD activity analysis at 550 nm. One enzyme unit corresponded to the amount of extract that resulted in 50% inhibition of xanthine reduction (U·mg^−1^ protein).

Measurement of catalase (CAT) activity was conducted as protocols of CAT assay kit (visible light; Jiancheng Corp., Nanjing, China). A total of 10% tissue homogenate was prepared with normal saline, and the supernatant was obtained by centrifugation for CAT activity analysis at OD405 nm. One enzyme unit was the amount of enzyme that decomposed 1 μmol H_2_O_2_ per mg of tissue protein per second (U·mg^−1^ protein).

The guaiacol method was used to detect the activity of peroxidase (POD) [42]. A total of 5 g of tissues were ground into homogenates at 0 °C in phosphate buffer (5 mL, pH 6.4), followed by centrifugation (4 °C, 20 min, 10,000× *g*) to collect the supernatant. A total of 2 mL of supernatant was removed into a 10 mL test tube with guaiacol (2.0 mL, 0.1%). After incubation at 37 °C for 5 min, 1 mL of hydrogen peroxide (0.1%) was added, and the OD470 values were immediately recorded every 30 s for 180 s. An increase of 0.01 in absorbance per min was recorded as one unit of POD activity (U·g^−1^ FW).

### 2.8. Assays of Defence-Related Enzyme Activity

The activity of phenylalanine ammonia-lyase (PAL) was detected by protocols of Assis et al. [43]. A total of 2 g of tissues were ground into homogenates at 0 °C in boric acid/borax buffer (4 mL, pH 7.4), containing 3% PVPP, 0.037 g of NaCl, and 0.137 mL of β-mercaptoethanol per 100 mL, and centrifuged at 4 °C for 20 min at 10,000× *g* to collect the supernatant. The reaction solution included 300 μL of supernatant, 600 μL of L-phenylalanine (0.02 mol/L), and 2.1 mL of boric acid/borax buffer (pH 8.7), followed by incubation at 37 °C for 60 min. Finally, the reaction was terminated with HCL (0.2 mL, 6.0 mol/L). No L-phenylalanine was added to the control system. An increase in the absorbance of 0.01 at 290 nm per hour was recorded as a unit of PAL activity (U·g^−1^ FW).

The catechol method was used to evaluate polyphenol oxidase (PPO) activity [42]. The preparation of enzyme extraction was the same as that for POD. Phosphate buffer (2.0 mL, pH 6.4) was mixed with the supernatant (0.5 mL) and placed at 37 °C for 5 min. A total of 2 mL of catechol (1%) was added to the reaction system, mixed well, and detected at 420 nm every 30 s for 3 min. An increase of 0.01 in the absorbance per min was equivalent to a unit of PPO activity (U·g^−1^ FW).

The activity of chitinase (CHI) and β-1, 3-glucanase was evaluated using the protocols of Yu et al. [44]. The extraction of crude enzyme solution was performed as follows. The grape sample (0.5 g) was added to 5 mL of disodium hydrogen phosphate-citric acid buffer (50 mmol/L, pH 5.0), homogenized at 0 °C and centrifuged (4 °C, 10,000× *g* for 20 min) to remove the supernatant for the analysis of CHI and β-L, 3-glucanase activities. A total of 0.5 mL of the supernatant, 1 mL of 0.4% (*w*/*v*) colloidal chitin, and 1.5 mL of disodium hydrogen phosphate-citric acid buffer (50 mmol/L, pH 5.0) were included in the CHI reaction mixture, followed by incubation at 37 °C for 60 min. After centrifugation one more time, 500 μL of the enzyme supernatant was collected and added to 10 μL of snailase (20%, *w*/*v*), followed by 60 min of incubation again at 37 °C. A total of 3 mL of 3,5-dinitrosalicylate acid (DNS, 2-fold dilution) was added to the reaction system, followed by heating for 6 min at 100 °C. Next, the mixture was diluted to 25 mL. The DNS method was conducted to determine the reducing sugars released. The OD530 value was detected, along with the reaction blanks. Snailase was not administered in the control group. One unit of chitinase activity was defined as the release of 1 μmol of N-acetylglucosamine (GlcNAc) per hour in the reaction mixture per mL (U·g^−1^ FW).

A total of 0.1 mL of the supernatant, 1 mL of 0.4% (*w*/*v*) laminarin, and 0.9 mL of disodium hydrogen phosphate-citric acid buffer (50 mmol/L, pH 5.0) were added in turn to the reaction mixture for the determination of β-1,3-glucanase activities. Following incubation (37 °C, 1 h), 3 mL of DNS (2-fold dilution) was used to terminate the reaction, followed by heating in boiled water for 10 min. Next, the mixture was diluted to 25 mL, and the OD530 values were detected. The reaction mixture was not incubated and served as blanks. The amount of glucose released was estimated using a standard curve prepared using glucose. One unit of β-1,3-glucanase activity corresponded to the release of 1 mg of glucose per hour in the reaction mixture per mL (U·g^−1^ FW).

### 2.9. Statistical Analysis

In all experiments of our study, a completely randomized design was conducted. Statistical evaluation was performed using SPSS version 22.0 (SPSS IBM, New York, NY, USA). Duncan’s multiple range test was used to analyse significant differences. The data were reported as the mean ± standard error (S.E.).

## 3. Results

### 3.1. Effects of Exogenous MT Treatment on Spore Germination and Mycelial Growth of B. cinerea

**Figure 1:**
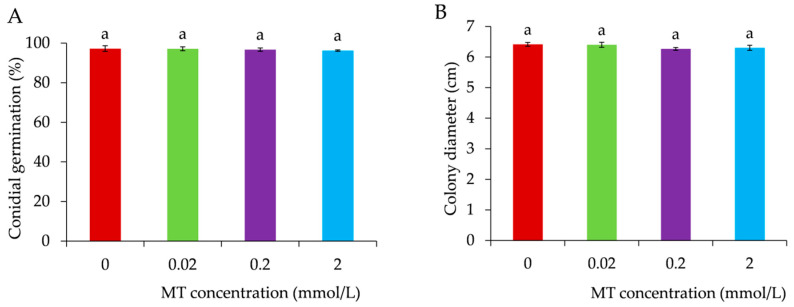
Effect of melatonin on conidial germination (**A**) and mycelial growth (**B**) of *B. cinerea*. Data represent means ± S.E. (*n* = 3). Duncan’s multiple range test was used to analyse significant differences in data, and letters above the bars represent differences at *p <* 0.05.

For *B. cinerea* infection, conidial germination is a critical step [45]. As shown in Figure 1A, with the addition of MT to the PDB liquid medium (0.02, 0.2, and 2 mmol/L), germination rates reduced slightly and decreased gradually from 97.1% to 96.2%, while the germination rate was 97.2% in PDB liquid medium. There was no significant difference (*p* > 0.05) among the groups. As seen in Figure 1B, the colony diameters of *B. cinerea* with different concentrations of MT were not significantly different among treatments at 4 d, all of which were above 6.00 cm. There was no significant difference compared to plates without MT (*p* > 0.05).

It has been reported that MT has no effect on the antifungal activity of *B. cinerea* in vitro but significantly decreases the grey mould infection in tomato plants [32,46]. Other research also found that 0.1–0.4 mmol/L MT did not detectably inhibit conidial germination and mycelial growth of *B. cinerea*, but MT had a significant effect on improving the activity of defence enzymes in fruits and inducing resistance to *B. cinerea* in apples [31]. A total of 0.2 mmol/L MT had little influence on antifungal activity against *Alternaria alternata* in vitro, but MT treatment increased the disease resistance of jujube fruit by regulating reactive oxygen species metabolism [47]. In our study, conidial germination or mycelial growth of *B. cinerea* in vitro were not significantly inhibited by different concentrations of exogenous MT, which agreed with previous studies. Disease inhibition of grapes may be related to the reduction of plant defence responses.

### 3.2. Effect of MT Treatment on the DI and DS in Grape Berries

**Figure 2:**
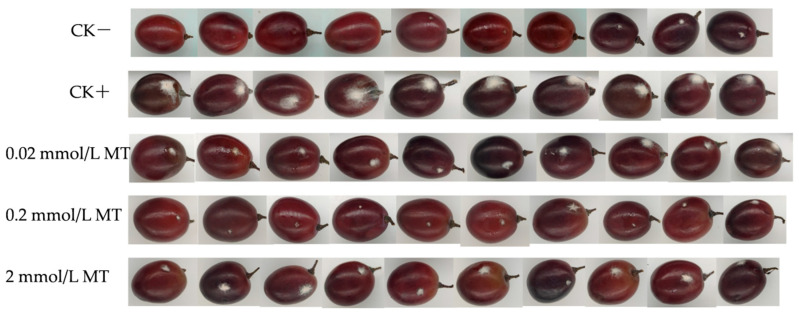
Effect of melatonin treatment on lesion development in infected “Muscat Hamburg” berries on day 3. Photographs depicting representative disease development were taken on day 3.

**Figure 3:**
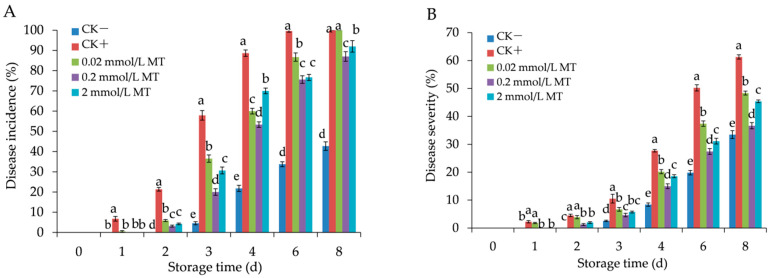
Effect of melatonin treatment on the disease incidence (**A**) and disease severity (**B**) in grape berries. Data represent means ± S.E. (*n* = 3). Duncan’s multiple range test was used to analyse significant differences in data, and letters above the bars represent differences at *p <* 0.05.

As shown in Figure 2, the lesion development in infected berries on day 3 was effectively controlled by different concentrations of MT, particularly at 0.2 mmol/L. With increasing fungal infection time, the DI and DS of the grapes displayed a gradual upward trend in all five groups (Figure 3A,B). The DI and DS of the MT treatment were significantly inhibited and presented at a lower level (*p <* 0.05) compared with those of the positive control groups from day 1 to day 6, with the most notable effect at 0.2 mmol/L. Meanwhile, DI and DS in the negative control group were at the lowest level. The results in Figure 2 and Figure 3 showed that MT treatment effectively reduced grey mould development in grapes.

### 3.3. Effect of MT Treatment on MDA and Cell Membrane Permeability in Grape Berries

**Figure 4:**
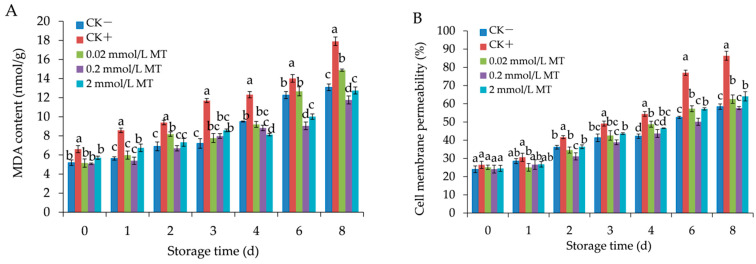
Effect of melatonin treatment on MDA (**A**) and cell membrane permeability (**B**) in grape berries. Data represent means ± S.E. (*n* = 3). Duncan’s multiple range test was used to analyse significant differences in data, and letters above the bars represent differences at *p <* 0.05.

As shown in Figure 4A,B, MDA content and cell membrane permeability in all groups continued to increase during the post-inoculation period, but those in the negative control had the slowest rate of rise. MDA content treated by different MT remained at lower values than those of the positive control until the 8th day (*p* < 0.05), especially at 0.2 mmol/L MT treatment (Figure 4A). The MDA content in the 0.2 mmol/L MT treatment was 34.29, 28.81, and 16.89%, respectively, lower than those of the positive control group, 2 mmol/L, 0.02 mmol/L treatment on day 8. As shown in Figure 4B, cell membrane permeability treated by MT was maintained at a lower level than those in the positive control group from day 2 to day 8 (*p* < 0.05). A total of 0.2 mmol/L MT treatment exhibited cell membrane permeability of 43.67, 50.12, and 57.64% on days 4–8, and remained significantly lower than those in the positive control (54.43, 77.01, and 86.36%) (*p* < 0.05). The studies showed that MT treatment could effectively reduce MDA accumulation and increasing of cell membrane permeability.

### 3.4. Effect of MT Treatment on Total phenolics and Flavonoids in Grape Berries

**Figure 5:**
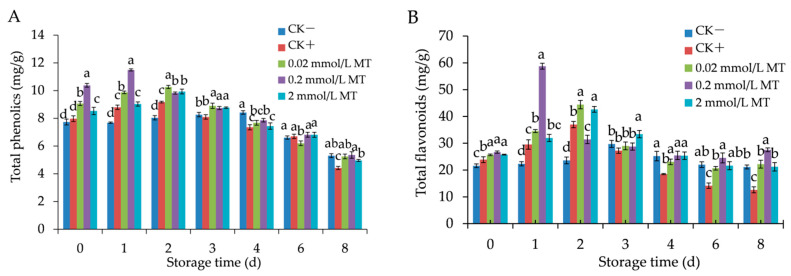
Effect of melatonin treatment on total phenolics (**A**) and flavonoids (**B**) in grape berries. Data represent means ± S.E. (*n* = 3). Duncan’s multiple range test was used to analyse significant differences in data, and letters above the bars represent differences at *p <* 0.05.

As visible in Figure 5A,B, the synthesis of total phenolics and flavonoids was remarkably increased in treatment and the positive control groups over the first 48 h and then decreased slightly after 48 h, except for those in negative control berries with a slight increase. The total phenolic content in the 0.2 mmol/L MT treatment group reached a peak on day 1, and those in the positive control, 0.02 mmol/L treatment, and 2 mmol/L treatment peaked on day 2 (Figure 5A). The peak value of the 0.2 mmol/L MT-treated group was 1.25, 1.12, and 1.16 times higher than those of the positive control, 0.02 mmol/L treatment, and 2 mmol/L treatment, respectively. Meanwhile, total flavonoid content in the 0.2 mmol/L MT treatment group peaked first (58.75 mg/g) on day 1, and the peak value was presented as a higher level compared with those of 0.02 mmol/L (44.42 mg/g), 2 mmol/L treatment (42.65 mg/g), and the positive control groups (36.96 mg/g) on day 2 (Figure 5B). These data indicated that different MT treatments could increase the synthesis of total phenolics and flavonoids over the fungal infection period, specifically the 0.2 mmol/L MT treatment.

### 3.5. Effects of MT Treatment on Antioxidant Enzyme Activities in Grape Berries

**Figure 6:**
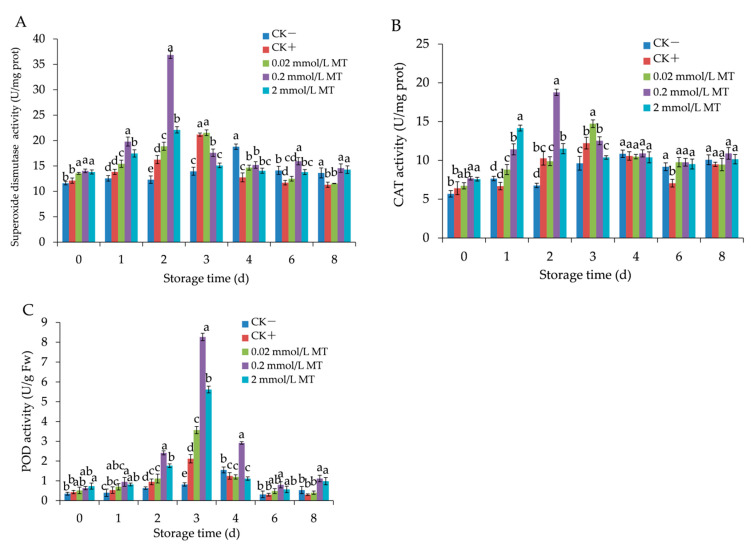
Effect of melatonin treatment on the activities of superoxide dismutase (**A**), catalase (**B**), and peroxidase (**C**) in grape berries. Data represent means ± S.E. (*n* = 3). Duncan’s multiple range test was used to analyse significant differences in data, and letters above the bars represent differences at *p <* 0.05.

SOD, CAT, and POD activities showed a unimodal trend with peaks in the five groups, and there were relatively highly different peak sizes and peak times between the experimental groups (Figure 6A–C).

The SOD activity reached the peak first on the 2nd day in the 0.2 and 2 mmol/L MT treatment groups (36.86, 22.11 U/mg, respectively); however, it reached the peak on the 3rd day in the positive control (21.18 U/mg) and 0.02 MT treatment (21.53 U/mg) groups, and last on the 4th day in the negative control (18.79 U/mg) group (Figure 6A). On the 1st to 8th day, the group treated with 0.2 mmol/L MT exhibited a marked increase in the SOD activity compared with other treated groups.

CAT also maintained a higher activity in the MT-treated groups than those in the control groups (Figure 6B). The 0.2 mmol/L MT treatment showed a higher peak value of CAT (18.77 U/mg) than those in the other groups (*p* < 0.05), and the value was 1.73, 1.53, 1.27, and 1.33 times greater than that in the negative control, positive control, 0.02 mmol/L, and 2 mmol/L MT treatment, respectively.

The POD activity reached the maximum in the positive control (2.11 U/g), 0.02 mmol/L (3.57 U/g), 0.2 mmol/L (8.27 U/g), and 2 mmol/L MT-treated groups (5.6 U/g), on the 3rd day, whereas in the negative control group, it reached the maximum value of 1.55 U/g on the 4th day (Figure 6C). MT treatment groups showed higher POD activities than the control groups, and the highest level was in the 0.2 mmol/L MT-treated group (*p* < 0.05).

### 3.6. Effects of MT Treatment on Defence-Related Enzyme Activities in Grape Berries

**Figure 7:**
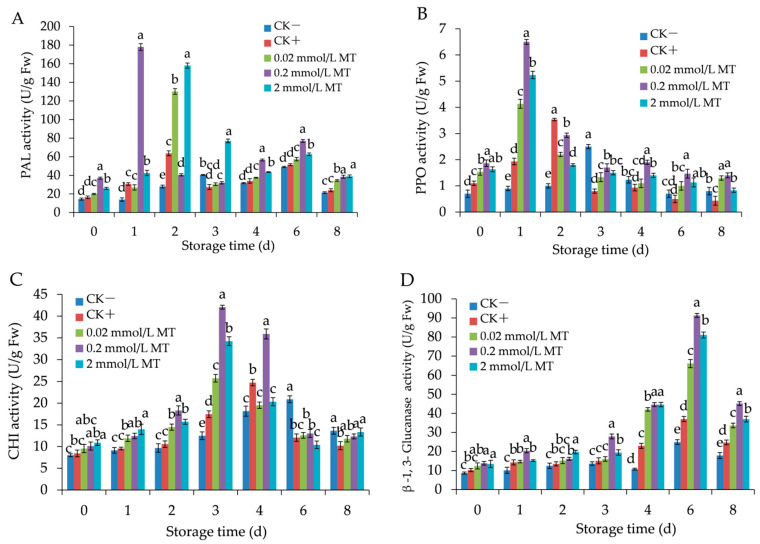
Effect of melatonin treatment on the activities of phenylalanine ammonia-lyase (**A**), polyphenol oxidase (**B**), chitinase (**C**), and β-1,3-glucanase (**D**) in grape berries. Data represent means ± S.E. (*n* = 3). Duncan’s multiple range test was used to analyse significant differences in data, and letters above the bars represent differences at *p <* 0.05.

PAL activity peaked on day 2 in the positive control (63.67 U/g), on day 1 in the 0.02, 0.2, and 2 mmol/L MT-treated groups (130.17, 178, and 158 U/g, respectively), decreased afterwards, and then increased again with peaks on day 6 (Figure 7A). However, the activity of PAL in the negative control group showed a small rise over the entire infection period. MT treatment significantly enhanced PAL activity (*p* < 0.05), specifically at a 0.2 mmol/L concentration.

PPO activity increased rapidly and reached maximal activity on day 1 in groups treated with 0.02, 0.2, and 2 mmol/L MT (4.13, 6.5, and 5.23 U/g, respectively). MT-treated groups presented higher PPO activities compared with the control groups, and PPO activity of 0.2 mmol/L MT-treated group was the highest level (Figure 7B).

CHI activity reached peaks on days 3, 4, and 6 in the MT-treated groups, positive control, and negative control, respectively, and then decreased thereafter (Figure 7C). Exogenous MT treatment significantly increased CHI activity (*p <* 0.05), and the most pronounced effect was recorded at 0.2 mmol/L concentration. β-1,3-glucanase activity peaked on day 6 in all groups tested, but MT treatment had a significant effect on enhancing the enzyme activity (*p <* 0.05) over the entire fungal infection period, particularly at a 0.2 mmol/L concentration (Figure 7D).

## 4. Discussion

Grey mould is the primary post-harvest disease in grapes [3]. Recently, MT has gained considerable attention because it could effectively induce plant resistance against pathogens. Our studies indicated that exogenous MT had a significant control effect on the post-harvest grey mould of grapes, but its prevention mechanism was not related to the bacteriostatic effect but rather induced fruit disease resistance. MT treatment group at 0.2 mmol/L had a pronounced effect compared with other MT-treated groups.

It has been reported that MT does not present obvious antifungal activity against *B. cinerea* [31,32,46] and *Alternaria alternate* [47] in vitro. However, MT treatment can reduce disease incidence and attenuate post-harvest decay of strawberries [29,30], apple [31], cherry tomato [32], and tomatoes [46]. Moreover, exogenous MT treatment at 0.25 mmol/L in litchi fruit could inhibit the incidence of litchi downy blight and expansion of the lesion inoculated with *P. litchi* [48]. Exogenous MT induced Malus resistance in Marssonina apple blotch [28]. MT treatment induced the jujube resistance against Alternaria rot [47]. Application of MT improved banana resistance to Fusarium wilt (*Fusarium oxysporum*) [49]. MT reduced the symptoms of downy mildew (*Pseudoperonospora cubensis*) in cucumber plants [50]. As far as we know, the effects of exogenous MT on the grey mould of table grapes have not been investigated in previous studies. Our studies found that exogenous MT could not significantly inhibit the growth and reproduction of *B. cinerea* in vitro, but it could significantly decrease disease incidence and attenuate disease severity, similar to previous results. Thus, the effect of MT in inhibiting grape grey mould might be due to improved disease resistance in grapes.

MDA content is an indication of membrane lipid peroxidation [51]. MDA content and cell membrane permeability can be used to reflect the membrane integrity of cells and the degree of membrane injury [52]. Our study indicated that exogenous MT treatment significantly inhibited MDA accumulation and decreased cell membrane permeability. Therefore, we deduced that MT treatment might alleviate the incidence of grey mould by slowing cell damage and retarding membrane lipid degradation.

After pathogen infection in grapes, ROS accumulation is strongly associated with the initial plant defence responses. Antioxidant defence systems in plants are activated to alleviate the stress generated by ROS [53,54]. Many studies have found that MT exhibits strong antioxidant capacity, which can scavenge ROS and increase ROS-scavenging enzyme activity, including SOD, CAT, and POD [31,46,48]. Among the enzymatic antioxidant defence systems, SOD dismutases O_2_^−^ to O_2_ and H_2_O_2_, whereas CAT and POD can convert H_2_O_2_ to H_2_O and O_2_ in cells [55,56]. Studies had shown that the upregulation of SOD, CAT, and POD activity in the ROS-scavenging system was observed when the resistance response of plants to biotic or abiotic stress occurred [47,57,58]. MT treatment can maintain a high scavenging capacity of ROS in cells, which was beneficial to alleviate the extent of membrane lipid peroxidation and preserve the cell membrane integrity [59,60]. Our study showed that SOD, CAT, and POD activities of grapes treated with exogenous MT or inoculated with *B. cinerea* were increased, but significant differences were observed among treatments for antioxidant activities. Exogenous MT treatment induced high SOD, CAT, and POD activities in the grapes during the early stages of infestation (days 1–3) and retained higher enzyme activities than the control groups during later stages (days 4–8), revealing that MT could activate the activities of ROS-scavenging enzymes and increase disease resistance in grapes.

PAL, PPO, and POD function as critical enzymes in the phenylpropanoid pathway, which have a positive effect on catalysing or participating in the biosynthesis of resistance-related substances [61,62]. PAL participates in the synthesis of resistant secondary metabolites such as total phenolics, flavonoids, and lignin to inhibit pathogen growth and strengthen the host cell structure [63]. These secondary metabolites possess marked bioactivities, and these compounds increased has been considered an indicator of plant defence responses [64]. PPO catalyses the oxidation of polyphenols into quinones in plants, which can inhibit pathogen growth and decrease the damage caused by pathogen infection in plants. POD was involved in phenol oxidation, suberisation, and lignification of host cells, and the wall-building processes can prevent the further extension of pathogenic agents [65]. Our study indicated that MT treatment had a significant effect on promoting PAL, POD, and PPO activities, increasing the synthesis of total phenols and flavonoids in grapes. These were in consonance with previous studies on cherry tomato and tomato of MT treatment [32,46]. The results also showed that these effects were associated with the increase of disease resistance against grey mould in grapes post-harvest.

CHI and β-1,3-glucanase performed essential functions in combating fungal infections. CHI can hydrolyze chitin in fungal cell walls, and β-1,3-glucanase can release oligosaccharides in fungal cell walls to induce a defence response [66,67]. Thus, CHI and β-1,3-glucanase activities increased can inhibit the growth of pathogenic fungi and stimulate and induce the appearance of potential defence responses in plants [68]. Previous studies have suggested that the activities of CHI and β-1,3-glucanase were remarkably promoted in fruit by MT treatment, which was related to the increase in disease resistance in fruits [28,32,46]. Our results also noted that CHI and β-1,3-glucanase activities were obviously increased after fungi infection, and the activity levels remained high during the entire trial compared with the control groups. This study indicated that MT could activate and maintain higher activities of CHI and β-1,3-glucanase, thereby possibly leading to the improvement of disease resistance in these grape plants.

## 5. Conclusions

In the present study, compared to control, exogenous MT-treated effectively decreased the disease incidence and alleviated the disease severity of grey mould in grapes; MDA content and cell membrane permeability maintained a lower level, and total phenolics and flavonoids presented with increasing content. The activities of enzymes including SOD, CAT, POD, PAL, PPO, CHI, and β-1,3-glucanase were remarkably promoted. The MT treatment at 0.2 mmol/L had the most noticeable effect. In conclusion, our study suggested that exogenous MT treatment directly activated defence responses by promoting ROS scavenging and defensive enzyme activities, increasing the content of resistance-related substances, and maintaining the membrane integrity of cells in grapes. Instead of inhibiting *B. cinerea* growth directly, exogenous MT treatment couldinduce disease resistance by priming defence mechanisms. However, further research on these mechanisms is required.

## Data Availability

The data presented is contained within the article.
